# Prevalence of Traumatic Dental Injuries and Their Associated Risk Factors in Eight- to 12-Year-Old Children in the State of Goa: A Cross-Sectional Study

**DOI:** 10.7759/cureus.94974

**Published:** 2025-10-20

**Authors:** Elaine S Barretto, Aswathy Sudarsanan, Dinesh F Swamy, Dorothy Cardozo

**Affiliations:** 1 Department of Pediatric and Preventive Dentistry, Goa Dental College and Hospital, Bambolim, IND

**Keywords:** permanent anterior teeth, prevalence, risk factors, school-going children, traumatic dental injuries (tdis)

## Abstract

Introduction: Traumatic dental injuries (TDIs) involving permanent anterior teeth are a prevalent type of dental injury in school-going children and can have a considerable impact on their physical health, psychological well-being, and social functioning. In the state of Goa, a study on TDI prevalence and its associated risk factors among children aged eight to 12 years has not been conducted.

Aim: This study aimed to determine the prevalence of TDIs and its association with various risk factors in eight- to 12-year-old children in the state of Goa.

Materials and methods: A cross‑sectional study was conducted on 2,732 school‑going children aged eight to 12 years. A detailed history and examination of 202 children who were identified with dental trauma was performed. Anterior dental trauma was assessed, and the data obtained were statistically analyzed.

Results: A prevalence of 202 out of 2,732 (7.39%) children was observed in the sample studied. TDIs in rural areas comprised 132 (65.34%) children. Single-tooth enamel fractures were most commonly seen. Left maxillary central incisors were mostly involved. About 80 (39.60%) subjects were unaware of their TDI incident. About 91 (45%) subjects had overjet <3 mm, three (1.5%) children showed anterior open bite, 168 (83.2%) children had Angle’s class I molar relation, and 194 (96%) children presented with competent lips. Among the subjects with trauma, only 25 (12.4 %) children sought treatment, while the remainder, 151 (74.8%) children, were unaware that they should seek treatment. Moreover, most of the children were unaware of the immediate management and first aid for TDI episodes.

Conclusion: This study provides valuable insights into the prevalence of TDIs among children in Goa, emphasizing the higher occurrence in rural areas, maxillary central incisors, and enamel fractures. Moreover, the study has found that only 25 (12.4%) children sought treatment for dental trauma, indicating the need for increased awareness and elaboration of prevention strategies for TDIs at the population level.

## Introduction

Traumatic dental injuries (TDIs) of permanent teeth are a significant health concern in children owing to their high prevalence rate, ranging from 4.1% to 58.6% [1] worldwide and 9% to 13% in India [2]. Despite the high prevalence of TDIs, treatment is often neglected due to a lack of awareness, high costs, and time constraints. TDIs are influenced by factors such as age, gender, and an overjet with inadequate lip coverage [3]. The current study was designed and conducted in Goa, as there was a lack of statistical data on the prevalence of TDIs and associated risk factors among eight- to 12-year-old children in the state.

## Materials and methods

The primary objective of this study was to determine the prevalence of TDIs, and the secondary objective was to evaluate the prevalence with its association with various risk factors in eight- to 12-year-old children in the state of Goa.

Study design and sample size

A cross-sectional study was carried out among 2,732 school children aged eight to 12 years, studying in the government and private schools of the 12 Talukas of Goa, to assess the prevalence of trauma to permanent anterior teeth. The estimated sample size was calculated based on the expected prevalence of a pilot study.

Formula used: n =  Z^2^P (1 - P) /d^2^ 

where n = the sample size, Z = the statistic corresponding to the level of confidence = 1.96, P = the expected prevalence or proportion = 7.71, and d = the precision or tolerated margin of error = 0.01.

Ethics statement

Ethical clearance was obtained from the Institutional Ethics Committee of Goa Dental College and Hospital, Bambolim, India (GDCH/IEC/I-2024(23)). Permission was obtained from the Directorate of Education, Porvorim, Goa, before conducting the study in school children.

Procedure

A random cluster sampling method was used for the study. The state of Goa is divided into 12 talukas. Each taluka formed a cluster, and one school per taluka was randomly selected by the lottery method. The study included all children aged eight to 12 years old, attending school on the day of the study, whose parents or caretakers were willing to provide consent. Children with missing permanent anterior teeth due to reasons other than trauma, those who did not cooperate for the oral examination with Frankl’s Behavior Rating Scale 1 and 2, and children with special health care needs were excluded from the study.

The examination was carried out between April and July 2024. After distribution of consent forms among the entire eligible class strength, a total of 2,732 children with consent forms and their parents/guardians were screened. Keeping in mind ethical considerations, all children received an awareness talk on oral health and TDI prevention and emergency management at the end of each data collection session.

The examination was done by a single calibrated dental surgeon (intra-examiner reliability was assessed during the pilot study, using Cohen’s kappa statistic, κ = 0.87) in natural light as per the WHO type III criteria (Inspection), and a detailed case history, followed by clinical examination, was taken from the children who reported trauma. Before the commencement of the intraoral examination, lip competency was observed in the resting position without the awareness of the child. The anterior dental trauma was assessed by the method used by Andreasen et al., consisting of visual assessment of tooth discoloration and dislocation of teeth [4]. Measurement of the maxillary overjet and assessment of molar relation and anterior open bite were made with the teeth in centric occlusion.

Children who reported trauma were asked about the treatment received after the TDI and were also assessed for their knowledge and awareness about TDI. TDIs in children may have occurred in a time frame of six months or longer, and sometimes the children may not recall the details of TDIs they experienced. This recall bias was anticipated, and separate codes were allotted for those who were unaware of the TDI and could not recall the TDI episode. The data were collected on the paper-based data collection form and statistically analyzed.

Statistical analysis

The data were statistically analyzed using IBM SPSS Statistics for Windows, Version 23.0 (released 2015, IBM Corp., Armonk, NY). Data analysis included descriptive statistics (frequency distribution and cross tabulation). The strength of association between the risk factors with the outcome was calculated by the Chi‑square test. The level of statistical significance was set at P < 0.05.

## Results

After the examination of 2,732 school children, it was observed that 202 (7.39 %) children had experienced TDIs to permanent anterior teeth. On comparing the association with gender, TDIs were found more in boys, about 119 out of 202 (58.9%), compared to girls, about 83 out of 202 (41.1%). TDIs in rural areas comprised 132 out of 202 (65.34%) children who reported with trauma.

**Figure 1:**
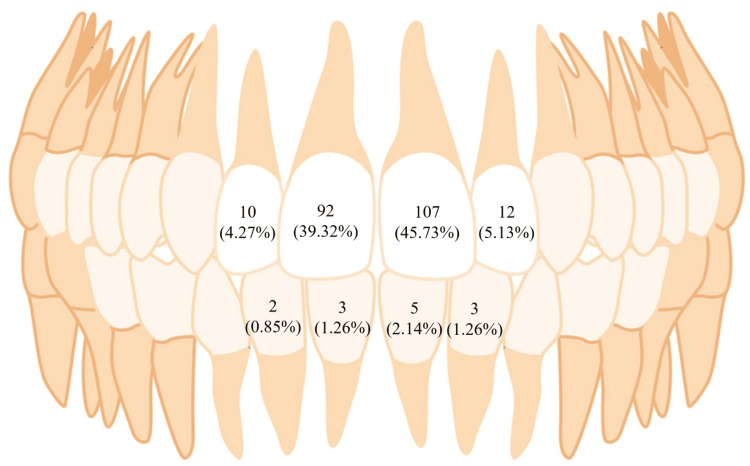
Distribution of traumatic dental injuries (TDIs): individual tooth involved.

Figure 1 shows the distribution of TDI based on the individual tooth involved. The most commonly affected teeth were maxillary incisors, with left central incisors (about 107 (45.73%) teeth) and right central incisors (about 92 (39.32%) teeth).

Table 1 shows the characteristics of TDIs. Among 202 subjects involved in trauma, single tooth enamel fracture, with 94 out of 202 (46.5%), was the most common type of injury (p < 0.0001), and 110 out of 202 (54.5%) children had only one TDI incident. About 80 (39.6%) of the subjects were not aware and could not recollect their TDI incident.

**Table 1:** Characteristics of traumatic dental injuries (TDIs).

Characteristics	n (%)
What kind of trauma?	
0 = No sign of injury	7 (3.5%)
2 = Enamel fracture	94 (46.5%)
3 = Enamel &dentine fracture	71 (35.1%)
4 = Pulpal involvement	3 (1.5%)
5 = Missing teeth due to trauma	1 (0.5%)
6 = Other damage	14 (6.9%)
How many times?	
Not aware	76 (37.6%)
1 time only	110 (54.5%)
2 times	12 (5.9%)
3 times	1 (0.5%)
4 times	1 (0.5%)
≥ 5 times	2(1.0%)
Where?	
Don’t know where it occurred	80 (39.6%)
At school– indoors	10 (5%)
At school – outdoors	6 (3.0%)
At home – indoors	50 (24.8%)
At home – outdoors	34 (16.8%)
At playground	10 (5%)
During sports	6 (3%)
During a medical emergency	4 (2%)
Miscellaneous	2 (1%)

Table 2 shows the association of risk factors of TDIs. Out of 202 children, 91 (45%) who had TDI had an overjet of less than 3 mm. The majority of the children, about 199 out of 202 (98.5%), who had trauma showed no anterior open bite. Competent lips were noted in 194 (96.0%) children with TDI. The prevalence of TDIs was found to be high in children with angle’s class I molar relation, about 168 out of 202 (83.2%) children.

**Table 2:** Association between traumatic dental injuries (TDIs) and their various risk factors.

Variables	n (%)	p
Lip competence		
Competent	194 (96%)	<0.0001
Incompetent	8 (4%)	
Overjet		
<3 mm	91 (45%)	0.4983
>3 mm	63 (31.2%)	
>5 mm	48 (23.8%)	
Anterior open bite		
Yes	199 (98.5%)	<0.0001
No	3 (1.5%)	
Molar relationship		
Class I	168 (83.2%)	<0.0001
Class II	4 (2%)	
Class II div 1	1 (0.5%)	
Class II div 2	2 (1%)	
Class III	6 (3%)	
End on	20 (9.9%)	
Others	1 (0.5%)	

Table 3 shows the treatment data of children reported with TDIs. Only 25 (12.4%) children received treatment, of which 15 (7.4%) children sought treatment within 24 hours, and among them, a majority of the children received medications and other treatments. About 177 (87.6%) children did not receive any treatment. The primary reason for not seeking treatment was their "unawareness of TDI” in about 151 (74.8%) children.

**Table 3:** Treatment data of children reported with traumatic dental injuries (TDIs).

Treatment	n (%)
Yes	25 (12.4%)
Sought treatment	
Within 30 minutes	1 (0.5%)
Within 1-2 hours	4 (2%)
Within 24 hours	15 (7.4%)
Next day	3 (1.5%)
After one week	2 (1.0%)
Treatment received	
Restoration	3 (1.5%)
Pulp treatment	2 (1.0%)
Prosthesis	1 (0.5%)
Extraction	0 (0.0%)
Medication	9 (4.5%)
Others	10 (5.0%)
No	177 (87.6%)
Reason for not seeking treatment	
Not aware	151 (74.8%)
Dentist not available	1 (0.5%)
Parents preoccupied with work	1 (0.5%)
Treatment not accessible	1 (0.5%)
Didn’t feel the need	22 (10.9%)
Other	2 (1.0%)

Table 4 shows the knowledge and awareness of children reported with trauma about TDIs and depicts the lack of proper awareness among them. Out of the total of 202, 175 (86.6%) children answered that a tooth that has broken or fallen out cannot be reattached or put back in place. About 140 (69.3%) children answered that the permanent tooth that has fallen out of the mouth due to trauma cannot be put back in place immediately by a dentist, and 197 (97.5%) children answered that it cannot be put back in place immediately by themselves. About 91 out of the 202 children (45%) said that tap water should be used to clean the tooth/fragments if they fell on the ground, and 132 out of 202 children (65.3%) replied that the tooth is to be held by the crown part. Regarding the storage of a tooth or fragments, which have fallen out or broken off, the majority of the children, about 104 out of 202 (51.5%), answered that it has to be stored in a plastic bag, cloth, or empty container (keeping it dry). This showed the lack of proper awareness among children about TDIs and associated emergencies.

**Table 4:** Knowledge and awareness of children reporting with trauma about traumatic dental injuries (TDIs).

	n (%)
Question 1: Are you aware that a tooth that has broken/fallen out can be reattached or put back in place?	
Yes	27 (13.4%)
No	175 (86.6%)
Question 2: Are you aware that a tooth that has fallen out can be put back in place?	
Yes	62 (30.7%)
No	140 (69.3%)
Question 3: If a (permanent) tooth has fallen out of the mouth due to trauma, would you put the tooth back in place immediately by yourself?	
Yes	5 (2.5%)
No	197 (97.5%)
Question 4: If the tooth or fragments have fallen on the ground, how will you clean it?	
Not to touch the tooth fragment/wipe with a handkerchief	49 (24.3%)
Use Alcohol/spirit	2 (1%)
Use Saliva	2 (1%)
Use an antiseptic solution	10 (5%)
Use tap water	91 (45%)
Use milk	21 (10.4%)
Others	27 (13.4%)
Question 5: How would you hold a tooth that has come out of the mouth?	
By the crown/white part	132 (65.3%)
By the root	27 (13.4%)
Not aware/don’t know	43 (21.3%)
Question 6: How would you store a tooth or fragments that have fallen out/broken off?	
Store it in a plastic bag, cloth, or empty container (Keeping it dry)	104 (51.5%)
In a container with tap water	45 (22.3%)
In a container with milk	17 (8.4%)
In a container with a storage solution	9 (4.5%)
In a container with saliva / inside a child’s mouth	2 (1%)
Place it back in the tooth position	0 (0%)
Others	25 (10%)

## Discussion

According to the findings of the present study, the prevalence of TDIs in the state of Goa in eight- to 12-year-old children was calculated as 7.39%. The TDI prevalence of similar age groups has been previously estimated by researchers in other parts of India. The findings of the present study, in comparison, were similar to those of Hegde et al. [5] in Navi Mumbai (7.3%); higher than the findings of David et al. [6] in Kerala (6%) and Vashisth et al. [7] in Kangra, Himachal Pradesh (5.12%); and lower than the findings of Panangipalli et al. [8] in Rajanagaram, Andhra Pradesh (12.1%) and Dharmani et al. [9] in Patiala, Punjab (11.4%). Prior research by Borkar et al. [10] in the same state, but for a younger age group of three to five years, found TDI prevalence to be as high as 16.1%.

In the current study, the prevalence of anterior tooth fractures in males compared to females was 1.43:1, aligning with findings from other studies that also noted a higher occurrence among males [5,11]. This may be due to their greater involvement in high-risk sports and physical activities, along with a higher likelihood of aggressive behavior, in contrast to more cautious and mature behavior seen in girls [5].

In the present study, the prevalence of TDIs observed in the rural areas compared to the urban areas was almost double (65.34% vs. 36.65%). This may be owing to the lack of education pertaining to TDI prevention and the lack of facilities in rural areas. However, as rural-urban infrastructure divides vary regionally, these findings are location-specific. This may explain the lack of statistically significant differences seen in TDI prevalence between rural and urban areas in the present study and in studies by Panangipalli et al. [8] (Rajanagaram, Andhra Pradesh) and Saraswathi et al. [12] (Rohtak, Haryana).

In the current study, the maxillary left central incisors were frequently affected (45.73%), followed by the maxillary right central incisors (39.32%) and maxillary left lateral incisors (5.13%). This corresponds with the results of earlier studies conducted by Andreasen and Andreasen [13], Nik-Hussein [14], and Ellis [15]. The increased probability of maxillary central incisors for TDIs is due to their early eruption, which exposes them to potential trauma for a longer period compared to lateral incisors. By contrast, mandibular incisors are relatively less susceptible to damage because the mandible's flexible attachment to the cranial base helps distribute impact forces more evenly.

In the present study, analysis of the type of injury shows that single-tooth enamel fractures were most common (46.19%) while the enamel-dentin fractures were next (37.39%). This may be because, in most cases, the trauma was not severe enough, resulting only in enamel fractures. The above-reported findings were, again, found to be in accordance with the findings of Nik-Hussein [14] and Bellamkonda et al. [16]. Crown fractures involving the enamel, dentin, and pulp were seen with lesser prevalence, while tooth avulsion was the least common injury sustained. In studies conducted by Kargul et al. [17] and Bellamkonda et al. [18], crown fracture involving enamel and dentin was reported to be the most prevalent injury sustained. In addition, most of the children (54.5%) in the current study had a TDI episode only once. Moreover, 39.6% of children were unaware of the cause of their dental trauma, possibly because the impact was so mild that they could not recall it. This aligns with the findings of Kaul et al. [19] and Bellamkonda et al. [18].

The association between increased incisal overjet and TDI has been extensively explored by numerous researchers, yet the literature reports on the correlation between the two factors are heterogeneous. According to a study conducted by Bellamkonda et al. [18], a statistically significant association was identified between overjet and the frequency of dental injuries, particularly noting a higher occurrence among children with an overjet exceeding 5.5 mm (28.62%). Moreover, in the meta-analysis conducted by Petti et al. [3], increased overjet accounted for up to 21.8% of TDIs worldwide. However, in the results of the current study, the majority of the children reported with an overjet <3 mm (45%), and there was no significant correlation seen between increasing overjets and TDIs. A similar conclusion was seen in Hegde et al.'s [5] study in Navi Mumbai. The present study found that 98.5% children had no anterior open bite and 96% had competent lips. Bellamkonda et al. [18] and Patel MC et al. [11] have suggested that in children, open bite and incompetent lips are important predisposing factors for TDIs.

In the present study, TDI was most frequently observed (83.2%) in children with Angle’s class I molar relation. A similar finding was reported by a study conducted by Govindarajan et al. [20], but the finding is in contradiction to the results of Bellamkonda et al. [16] and Panangipalli et al. [8], who found Angle’s class II malocclusion to be most frequently observed with TDIs. Hence, these authors suggested that correcting malocclusion and improving lip closure may help reduce the risk of anterior tooth fractures.

Only 12.4% of children sought treatment for dental trauma; among them, only 7.4% sought treatment within 24 hours. Most of the children received medications and other treatments, like observation and follow-up. About three-fourths of those who reported with TDIs were unaware of the treatments for TDIs. This could be because most of the TDIs were single-tooth enamel fractures, and the majority were unaware of TDIs in the current study. A similar study conducted by Khandelwal et. al [21] reported that only 2.5% of the cases of TDIs had undergone treatment. This indicates a low level of awareness regarding the importance of treatment and facilities available for the treatment of TDIs.

The majority of children (86.6%) believed that a broken tooth could not be reattached. In addition, 69.3% assumed that an avulsed permanent tooth due to trauma could not be immediately repositioned by a dentist, while 97.5% believed they could not do it themselves. Singh B et al. [22] reported that, when the children were asked how they handled a broken tooth segment, the most common response (34.5%) was to leave it on the ground. This may be attributed to a lack of awareness or a careless attitude toward oral health.

In the present study, most of the children (45%) responded that if the avulsed tooth is contaminated, it should be cleaned with tap water. A significant majority replied that the tooth has to be held by the crown part, indicating the growing awareness among the population about emergency care of dental trauma. However, regarding the storage of a tooth or its fragments, which have fallen out/broken off, most of the children (51.5%) replied that it has to be stored in a plastic bag, cloth, or empty container, i.e., ‘dry storage’. This indicated the need for more awareness among the children about TDIs. One of the objectives of epidemiological studies should be to educate and motivate children to seek appropriate treatment, prevention strategies, and immediate care for TDIs. More awareness results in better prevention and treatment outcomes of TDIs.

In the current study, the diagnosis of dental trauma was based solely on clinical examination, without the use of radiographic imaging due to logistical limitations. This major limitation may have resulted in the omission of certain injuries, such as root fractures, which are not detectable without radiographs. Due to the cross-sectional design of this study, only injuries visible on the day of examination were recorded, and injuries to the supporting structures of the teeth may be underreported, as they can heal without leaving any visible signs. Consequently, the actual prevalence of such injuries may be higher than reported. Also, the majority of children were unaware of their TDIs, which could have resulted in the underreporting of associated factors responsible for such injuries. Future studies should incorporate longitudinal designs and radiographic assessments to provide more accurate data on the long-term impact of dental trauma.

## Conclusions

This study provides valuable insights into the prevalence, patterns, and awareness of TDIs among children in Goa, emphasizing the higher occurrence in rural areas, the vulnerability of maxillary central incisors, and the predominance of enamel fractures. While no strong correlation was found between increased overjet, anterior open bite, or lip incompetence with TDIs, further research is required to explore these associations comprehensively. A significant gap in awareness regarding the management and treatment of dental trauma was observed, highlighting the need for targeted educational initiatives about prevention, emergency management, and timely treatment of TDIs, ultimately improving oral health outcomes in children.
